# Supplementary material for: Impaired Ca2+ Sensitivity of a Novel GCAP1 Variant Causes Cone Dystrophy and Leads to Abnormal Synaptic Transmission Between Photoreceptors and Bipolar Cells
Source: Int J Mol Sci. 2021 Apr 14;22(8):4030. doi: 10.3390/ijms22084030 (PMC8070792; doi:10.3390/ijms22084030)
Supplement: Supplementary file 1 [file ijms-22-04030-s001.pdf]

International Journal of *Molecular Sciences*

MDPI

Supplementary Materials

Impaired Ca^{2+} sensitivity of a novel GCAP1 variant causes cone dystrophy and leads to abnormal synaptic trans-mission be-tween photoreceptors and bipolar cells

Valerio Marino, Giuditta Dal Cortivo, Paolo Enrico Maltese, Giorgio Placidi, Elisa De Siena, Benedetto Falsini, Matteo Bertelli and Daniele Dell'Orco

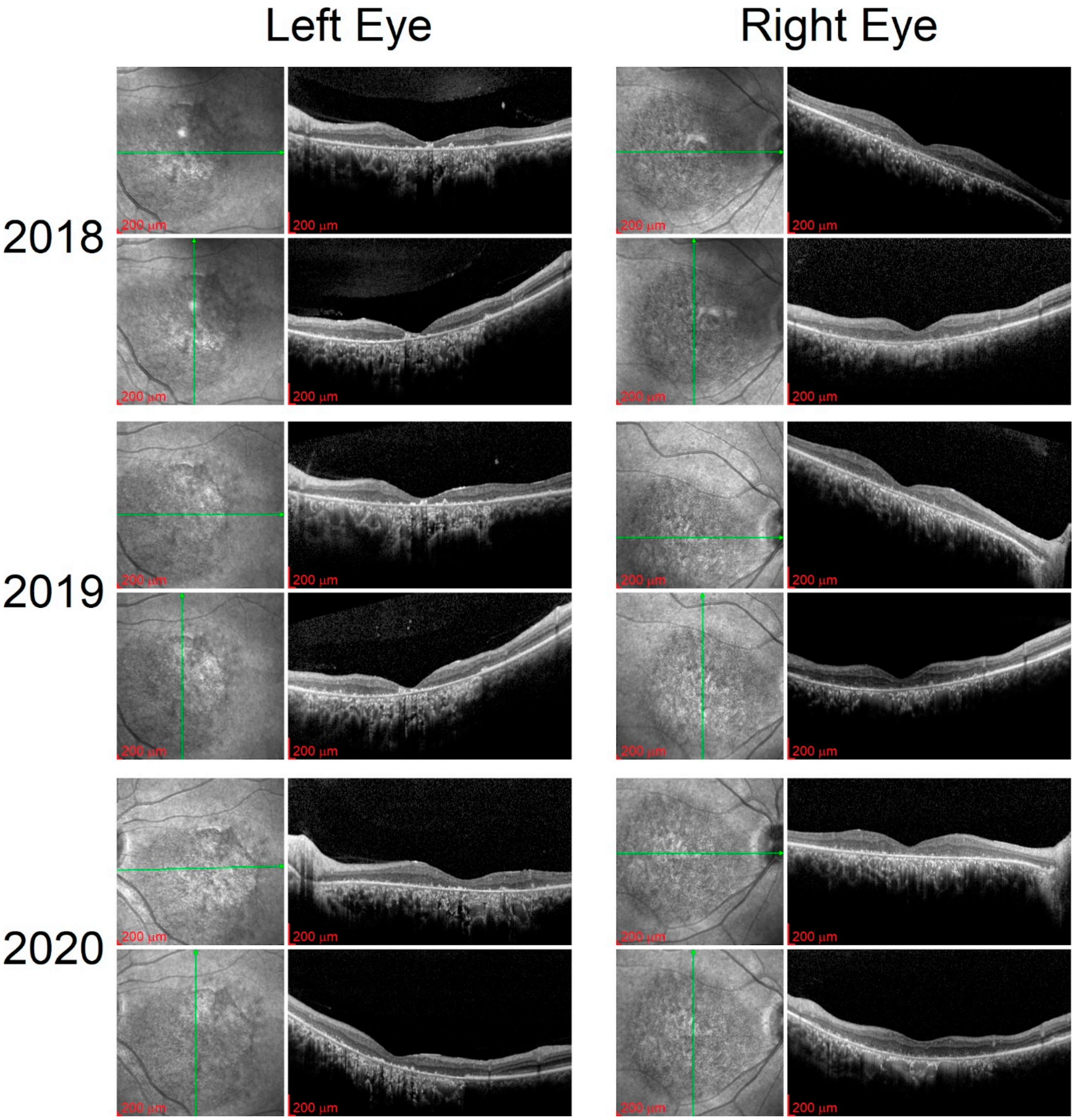

Figure S1. Fundus autofluorescence and optical coherence tomography recorded in the study patient during a three-year follow up.

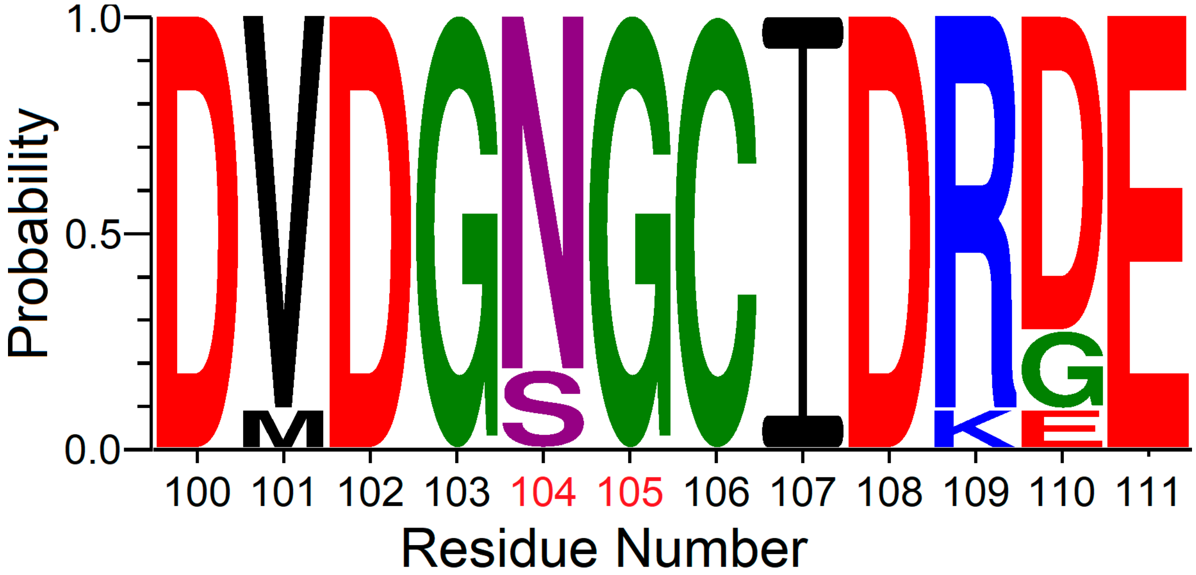

Figure S2. Logo representation [1,2] of the consensus sequence of GCAP1 EF-hand 3 motif in 11 homologous sequences from 10 vertebrate species (HomoloGene ID 353): Homo sapiens, Pan troglodytes, Macaca mulatta, Canis lupus, Bos taurus, Mus musculus, Rattus Norvegicus, Gallus gallus, Xenopus tropicalis and Danio rerio. Residues are colored according to their physicochemical properties: acidic (red), hydrophobic (black), special (green), polar (violet), basic (blue). The residue number of the two substitutions is represented in red.

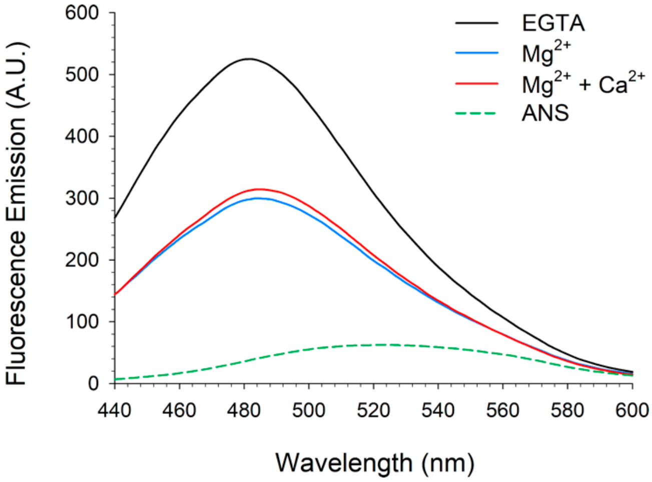

Figure S3. Emission fluorescence spectra of 30 µM ANS (green dashed line) and ANS incubated with 2 µM GCAP1 N104K-G105R in the presence of 500 µM EGTA (black) and after serial additions of 1 mM Mg^{2+} (blue) and 1 mM Ca^{2+} (red).

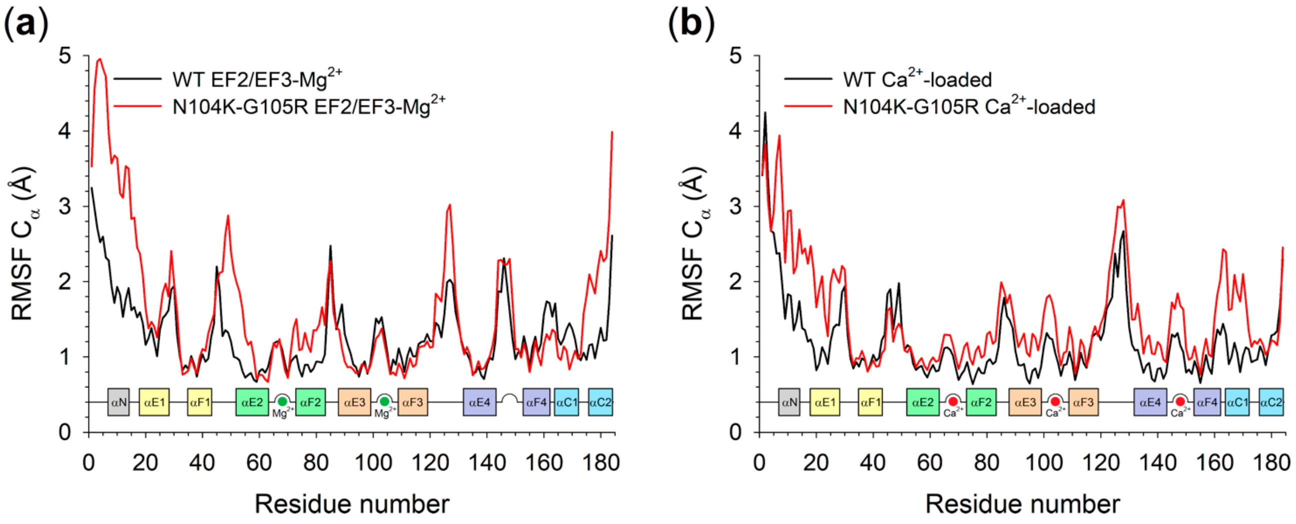

Figure S4. Cα-RMSF profiles (calculated over 300 ns MD simulations) of (a) EF2/EF3 Mg^{2+}-bound GCAP1 WT (black) and N104K-G105R variants and (b) Ca^{2+}-loaded GCAP1 WT (black) and N104K-G105R variants. Inset shows secondary structure elements with the same coloring scheme as Figure 4A and ion binding loops, where Mg^{2+} and Ca^{2+} ions are represented as green and red spheres, respectively.

Table 1. Regulation of GC1 and GC2 by GCAP1 variants.

Variant	X-fold GC1[a]	X-fold GC2[a]
**WT**	37.4 ± 11.6	10.8 ± 5.3
**N104K-G105R**	0.47 ± 0.07	0.6 ± 0.2

[a]X-fold is calculated as $([cGMP]_{low\_Ca} - [cGMP]_{high\_Ca}) / [cGMP]_{high\_Ca}$. ± std. dev.

Table 2. RMSF of cations bound to GCAP1 variants.

		RMSF (Å)[a]		
**Variant**	**Cation**	**EF2**	**EF3**	**EF4**
**WT**	**Mg^{2+}**	0.918	1.099	
	**Ca^{2+}**	0.834	1.002	0.955
**N104K-G105R**	**Mg^{2+}**	0.887	0.952	
	**Ca^{2+}**	1.008	1.305	1.236

[a] Root-Mean Square Fluctuation of ions calculated as the time-averaged Root-Mean Square Deviation from the average position over 300 ns MD simulations.

Reference

1. Crooks, G.E.; Hon, G.; Chandonia, J.M.; Brenner, S.E. WebLogo: A sequence logo generator. *Genome Res.* **2004**, *14*, 1188–1190, doi:10.1101/gr.849004.
2. Schneider, T.D.; Stephens, R.M. Sequence logos: A new way to display consensus sequences. *Nucleic Acids Res.* **1990**, *18*, 6097–6100, doi:10.1093/nar/18.20.6097.
